# Coronary artery bypass grafts and diagnosis related groups: patient classification and hospital reimbursement in 10 European countries

**DOI:** 10.1186/s13561-014-0004-8

**Published:** 2014-04-10

**Authors:** James Gaughan, Conrad Kobel

**Affiliations:** 1 Centre for Health Economics, University of York, York, UK; 2 Australian Health Services Research Institute, University of Wollongong, Wollongong, Australia; 3 Department of Medical Statistics, Informatics and Health Economics, Innsbruck Medical University, Innsbruck, Austria

**Keywords:** Diagnosis-related groups, DRGs, Hospital financing, Coronary artery bypass graft, CABG, Reimbursement

## Abstract

**Background:**

The prospective reimbursement of hospitals through the grouping of patients into a finite number of categories (Diagnosis Related Groups, DRGs), is common to many European countries. However, the specific categories used vary greatly across countries, using different characteristics to define group boundaries and thus those characteristics which result in different payments for treatment. In order to assist in the construction and modification of national DRG systems, this study analyses the DRG systems of 10 European countries.

**Aims:**

To compare the characteristics used to categorise patients receiving a coronary artery bypass graft (CABG) surgery into DRGs. Further, to compare the structure into which DRGs are placed and the relative price paid for patients across Europe.

**Method:**

Patients with a procedure of CABG surgery are analysed from Austria, England, Estonia, Finland, France, Germany, Ireland, Poland, Spain and Sweden. Diagrammatic algorithms of DRG structures are presented for each country. The price in Euros of seven typical case vignettes, each made up of a set of a hypothetical patient’s characteristics, is also analysed for each country. In order to enable comparisons across countries the simplest case (index vignette) is taken as baseline and relative price levels are calculated for the other six vignettes, each representing patients with different combinations of procedures and comorbidities.

**Results:**

European DRG payment structures for CABG surgery vary in terms of the number of different DRGs used and the types of distinctions which define patient categorisation. Based on the payments given to hospitals in different countries, the most resource intensive patient, relative to the index vignette, ranges in magnitude from 1.37 in Poland to 2.82 in Ireland. There is also considerable variation in how much different systems pay for particular circumstances, such as the occurrence of catheterisation or presence of comorbidity.

**Conclusion:**

Past experience of the construction of DRG systems for CABG patients demonstrates the variety of options available. It also highlights the importance of updating systems as frequently as possible, to incentivise best practice.

## Background

### DRG background

Diagnosis Related Group (DRG) systems use clinically meaningful diagnostic and procedural characteristics to categorise patients into a manageable number of resource homogenous groups. Each group is treated like a product, so a hospital may be viewed as a firm producing multiple products [1]. While the original intention behind DRGs was to develop a tool to measure resource utilisation, DRGs soon became the foundation for a number of applications [2]. One common use of DRGs is as a reimbursement tool. Each group is assigned a value, which reflects the average cost of patients within the DRG [3]. This incentivises efficient use of resources as excess payments are retained and costs paid by the hospital [4]. DRGs are also used to measure hospital performance, by removing the impact of case mix differences from observable outputs [5]. Finally, such a reimbursement system can financially incentivise best practice, by paying a higher price when additional resources are needed (in the form of tests, procedure type or length of stay), but not doing so when such additions are not optimal. Thus, a major aim of DRG systems is forming group boundaries that are associated with legitimate differences in cost/resource consumption when the most appropriate treatment is used. Failure to adequately map reimbursement to legitimate cost, either by attaching different reimbursement to patients of similar cost or by grouping patients with large differences to the same tariff, results in a mismatch between the profit maximising treatment and the most appropriate treatment. Such a mismatch could result in patients receiving inappropriate treatment or the provider of treatment being financially penalised for giving clinically optimal care.

Two case group types provide particular challenges in constructing and maintaining a DRG system. First, more complex cases with comorbidities have greater potential heterogeneity, forcing a system to have a larger number of sparsely populated DRGs or a smaller number of more heterogeneous categories. Second, when advances in medical research are faster, the ideal treatment approach changes more frequently. If a DRG system does not keep pace with advances, it could restrict the dissemination of new approaches by financially incentivising outdated methods.

Determining characteristics accurately is most important when the budgetary impact of a subset of DRGs is large, due to the average expense of cases or the number of patients concerned. The cost of inaccuracy to performance and best practice is greatest in these cases. As such, regular updating of DRG systems is vital for meeting the objectives of a DRG system.

### Clinical background

Ischemic heart disease (IHD) is the most frequent cause of death worldwide. In 2008, 12.8% of global deaths were attributable to IHD and 15.6% in high income countries [6]. Coronary artery bypass graft (CABG) surgery is one of the two main surgical treatment options for IHD. This revascularisation procedure diverts the flow of blood around blocked or restricted vessels supplying blood to the heart. Alternatives such as Percutaneous Transluminal Coronary Angioplasty (PTCA), which widens the restricted vessel, are generally preferred for single vessel and anatomically uncomplicated cases [7,8]. On the other hand, CABG procedures are increasingly performed on older and more moribund patients [9]. This trend suggests that the reimbursement of CABG procedures in the context of other surgical procedures such as valve replacements and the presence of comorbidities will become all the more critical as heterogeneity of patients and the associated costs increase.

### Our contribution

In this study, we analyse the differences in patient classification in ten European countries (Austria, England, Estonia, Finland, France, Germany, Ireland, Poland, Spain and Sweden)^a^ and the outcome of this on reimbursement. The comparison of classification characteristics is facilitated by seven hospitalisations for CABG surgery of differing complexity (case vignettes), which also highlight the impact of variation in the relative reimbursement of these vignettes. This approach provides an overview of past experience and some initial comparisons of variation in approach upon the reimbursement of hospitals for the treatment of patients requiring CABG surgery.

This work is performed within the framework of the EuroDRG^b^ project, which looks at hospitalisations for ten conditions, analysing the ability of different DRG systems to account for patient characteristics, as well as analysing the impact of a variety of patient characteristics upon the cost or length of stay of patients [10,11]. In this article we focus on CABG surgery, as one of the most frequently performed major surgical procedures and a procedure with many variations in approach.

## Methods

### Data

Researchers obtained patient level data at national or regional level, containing core information on diagnoses, procedures and DRG for each patient, in order to identify agreed conditions. A description of the sources of data used is given in Table 1. From this core information, analysis is performed on two characteristics of DRG systems. First, the structural nature of the DRG system, highlighting the hierarchy of decision making for patient classification. Second, the specific DRGs and values assigned to a set of case vignettes, to investigate the impact of variation in the allocation of patients and values to DRGs upon reimbursement.

**Table 1:** Sources of data

**Country**	**Source of data**	**Year**
**Austria**	Performance-oriented Hospital Financing Framework Database	2008
	Private Hospitals Financing Fund Database	2008
**England**	Hospital Episode Statistics (HES)	2007/08
	National Health Service Reference Costs	2007/08
	CHE Trust Database	2007/08
**Estonia**	Estonian Health Insurance Fund (EHIF) Database	2008
**Finland**	Hospital Discharge Register (hospitals of Helsinki and Uusimaa)	2008
**France**	National Hospital Cost Study (ENCC; representative sample)	2007
	Hospital Inpatient Activity Database (PMSI MCO; all hospitals)	2008
**Germany**	Research database based on patient-level data according to §21 Hospital Remuneration Act (KHEntG) and national G-DRG cost accounting standards by the Institute for the Hospital Remuneration System (InEK)	2008
**Ireland**	Hospital In-Patient Enquiry (HIPE)	2008
**Poland**	Central Register of Healthcare Services and Reimbursements	2009
**Spain (Catalonia)**	Public Hospital Network of Catalonia	2008
	Spanish Network of Hospital Costs	2008/09
**Sweden**	National Case Costing Database	2008

### Patient classification systems

**Figure 1:**
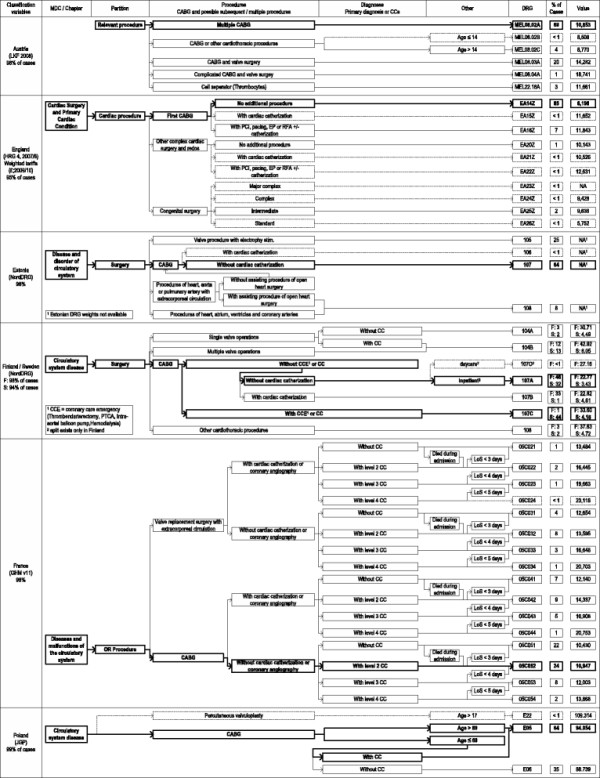
Grouping structure.

**Figure 2:**
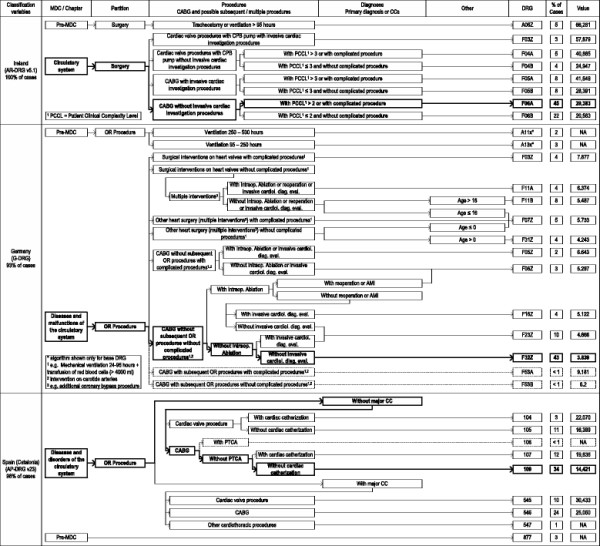
Grouping structure.

Within the EuroDRG project, we define a CABG case as admission to hospital of a patient who undergoes a coronary artery bypass graft surgery (procedure code 36.1 in ICD-9-CM^c^). Patients aged under one year are excluded from the analysis. In order to compare structural differences in patient classification, the CABG cases conforming to the definition described and the DRGs to which these patients are categorised, is presented graphically in Figures 1 and 2. These figures show the grouping hierarchy and the final proportions of CABG cases in each DRG. Analogously to the other works of the EuroDRG project, particular attention is placed on DRGs which contain more than 1% of CABG cases [11]. However, all DRGs which elucidate the overall structure are also included, even if less than 1% of CABG cases are assigned to them. In the figures, these DRGs are marked with dashed lines, while the DRGs with the highest proportion of CABG cases are highlighted bold.

DRG structures are considered in two ways. First, the number of DRGs and specific characteristics used to allocate a particular case to one DRG or another. A larger number of DRGs allows for a more nuanced allocation of patients at the expense of complexity and greater potential for unintended incentives, where clinically and financially optimal treatment diverges. Further, the presence or absence of any given characteristic in a DRG structure generally represents the presence or absence of a differential in reimbursement on the basis of that characteristic. Alternative approaches to reimbursement such as surcharges are also discussed. Table 2 gives a summary of the characteristics used by different DRG systems to allocate cases to DRGs.

**Table 2:** DRG split variables

**Country**	**Age**	**LoS**	**Comorbidities/complications**	**Catheterisation**	**Valve procedure**	**Death**	**First CABG**
Austria	---	---	x	---	x	---	---
England	---	---	---	x	x	---	x
Estonia	---	---	---	x	x	---	---
Finland	---	---	x	x	x	---	---
France	---	x	x	x	---	x	---
Germany	x	---	x	x	---	---	---
Ireland	---	---	x	---	---	---	---
Poland	---	---	x	---	---	---	---
Spain	---	---	x	x	x	---	---
Sweden	---	---	x	x	x	---	---

Source: [10] Table 1.

Second, the hierarchy of DRG structures are considered. The importance placed on a characteristic depends upon its place in the overall structure and it is important to note that not all structures are symmetrical. For example, catheterisation is a diagnostic test which may be present in a structure. However, the impact and particularly incentives attached to this test would differ if it was the first characteristic considered compared to a scenario where it is only considered for a subset of CABG cases, such as less complex cases.

### CABG case vignettes

One of the major methodological issues which arises when quantitatively comparing DRG systems across countries, is variation in purchasing power between nations. Therefore, even after adjusting for currency differences, some of the variation in observed price will be due to variation in the cost of labour and cost of living. Further, in comparing DRG prices, there is variation in the parts of cost included in a DRG tariff, see [12] for details. In order to draw comparisons between countries, seven case vignettes are used. Of these, the simplest one is used as the index vignette. The relative prices for all other vignettes are calculated by dividing the absolute prices by the price of the index vignette. So the relative price of the index vignette equals 1 for all countries.

The *index vignette* contains the simplest and most prevalent CABG DRG in all ten European systems studied. The specific case is a 65 year old patient, who undergoes a bypass of a single vessel without any additional procedures or any comorbidity. The length of stay (LoS) is 12 days. As such, all the relative prices calculated, compare to the DRG to which this case is allocated.

*Vignette 1* involves a multiple vessel bypass (specifically 3 vessels) and the use of catheterisation on a patient, also 10 years older than the index vignette. A valve procedure is performed along with CABG surgery, with the use of catheterisation, in *case vignette 2*. This patient also has a shorter LoS than the index vignette at nine days and the stay ends in death. *Vignette 3* is most similar to vignette 1, with an additional three days of LoS and diagnoses of diabetes, dilated cardiomyopathy and cerebral infarction (Stroke). *Vignette 4* combines multiple vessel bypass with a valve procedure but without catheterisation. The patient also has chronic renal failure. Vignette 4 also has a longer LoS than the index vignette (18 days). *Vignette 5* includes a secondary diagnosis with valve procedure and catheterisation. The diagnosis is of atrial fibrillation. This patient has the longest LoS at 20 days. Finally, *vignette 6* involves the same procedures as vignette 4, performed on a 75 year old patient, also diagnosed with acute transmural myocardial infarction of anterior wall. Another distinguishing feature of this vignette is that the case ends in death after a two day inpatient stay. The definition of the vignettes given in ICD-9-CM and ICD-10^d^ are presented in Table 3.

**Table 3:** Case vignette specifications

	**ICD-9-CM procedure codes**	**Vessels**	**Primary diagnosis**	**Secondary diagnoses**	**Catherisation**	**Valve surgery**	**Age**	**Death during admission**	**LoS**
Index	36.11	1	I25.1	---	No	No	65	No	12
Case 1	36.13	3	I25.1	---	Yes	No	75	No	12
Case 2	36.11	1	I25.1	---	Yes	Yes	65	Yes	9
Case 3	36.11	1	I25.1	E11.8, I42.0, I69.3	Yes	No	75	No	15
Case 4	36.13	3	I25.1	N18.0	No	Yes	65	No	18
Case 5	36.11	1	I25.1	I48	Yes	Yes	75	No	20
Case 6	36.13	3	I35.0	I21.0	No	Yes	75	Yes	2

Notes:

I25.1 - Atherosclerotic heart disease.

I35.0 - Aortic (valve) stenosis.

E11.8 - Non-insulin-dependent diabetes mellitus with unspecified complications.

I10.0 - Essential (primary) hypertension.

I21.0 - Acute transmural myocardial infarction of anterior wall.

I42.0 - Dilated cardiomyopathy.

I48 - Atrial fibrillation and flutter.

I69.3 - Sequelae of cerebral infarction.

N18.0 - Chronic renal failure: End-stage renal disease.

The value of a DRG is either its assigned cost weight or price, depending on the particular DRG system [3]. Where DRGs are attached to weights (Estonia, Finland, Germany, Ireland Spain and Sweden) or scores (Austria and Poland), rather than prices (England and France), national conversion systems to reflect average cost of the DRG are used. The reimbursement price of each vignette is then divided by the reimbursement price for the index vignette. This provides the relative value placed on each vignette by each DRG system, compared to the index vignette. However, it should be noted here that as DRGs represent average price and are of varying sizes, so are the values attached to the index vignette, as that of the DRG to which the index vignette is assigned, retains that variability.

From this process, two closely related features are considered across countries. First, the DRG to which each vignette is allocated. This serves to highlight the impact of the choice of different characteristics to define DRGs and the hierarchy of those characteristics. Such differences result in some different cases being treated as the same in some systems but not others.

Second, the specific relative reimbursement levels for the same vignette are considered. This shows the different levels of importance placed on particular characteristics between systems and within them, since the same characteristic may result in a different change in reimbursement in different scenarios. Considering relative reimbursement also directly accounts for adjustments made in payment that are not through a change of DRG. This is important as it is final reimbursement which drives incentives, rather than a DRG allocation.

## Results

### Grouping hierarchy

Figures 1 and 2 highlight differences in the number and types of characteristics used to group patients, as well as the degree of division within each. Differences exist in the number of DRGs used to categorise CABG cases. This ranges from two (Poland) to 16 (France), with the majority of systems analysed having between four and eight groups. There is also variation in the concentration of patients within DRGs. The proportion of CABG cases in the most populous DRG category of each country ranges from 24% (France) to 85% (England) and the total proportion of CABG patients covered by DRGs containing at least 1% of the total sample ranges from 92% (Germany) to 100% (Ireland). For better comparison, the presentation of the grouping hierarchy and the terms used to describe DRGs are to some extent harmonised.

Generally the grouping process follows the same steps across the studied systems. The patient’s principal diagnosis determines the Major Diagnostic Category (MDC)^e^, except in Austria where no such concept is used. The MDC for our CABG cases concerns the circulatory system, except in Germany, Ireland and Spain, where some extreme resource consuming cases (long-lasting mechanical ventilation) are grouped into the Pre-MDC, designed for this purpose.

The second step includes the decision whether the treatment is considered a surgery, sometimes also called operating room (OR) procedure, or if the treatment is not. All CABG cases are considered as surgery and hence are grouped into the corresponding partition. Only the Polish system does not make this distinction.

Next, the actual treatments become relevant. Generally, the systems distinguish between two different scenarios. The first is a valve surgery together with a CABG during the same hospitalisation. In this case, the valve surgery becomes the dominant grouping criteria. The second scenario is a “classical” CABG surgery (without valve surgery). Especially in the latter case, systems further differentiate the types of CABGs, e.g. first (England), multiple (Austria) or complex (Austria and Germany) CABGs. Additional accompanying treatments are used as split variables in a number of countries such as cardiac catheterisation (England, Estonia, Finland, France, Ireland, Germany and Spain), the most frequently used characteristic for CABG cases.

To determine the severity of a case, “Complications or Comorbidities” (CC) based on secondary diagnoses are used as grouping criteria in some countries (Finland, France, Poland, Spain and Sweden). This ranges from a single dichotomy of CCs in Poland, Finland and Sweden to four levels of CCs in France. In Ireland, a so-called Patient Clinical Complexity Level (PCCL) is used to determine the cumulative effect of all diagnoses reported for a particular patient. Instead of CCs, in Germany the primary diagnosis Acute Myocardial Infarction (AMI) is used in one part of the structure. Instead or in addition to CC splits, other criteria can be used as well. Age is used in Austria, Germany and Poland. Length of stay, particularly as a cut off value, or whether a patient dies in hospital determines the DRG in France; and in Finland, the type of admission is used once. It will be seen below that while each DRG has an attached reimbursement, it is not always the only determinant of reimbursement. Most commonly, reductions or surcharges apply on a per diem basis for short-stay or long-stay outliers.

### Case vignettes

**Figure 3:**
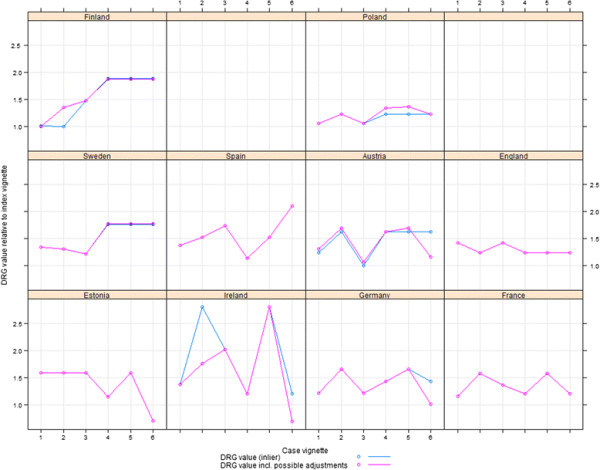
Relative reimbursement of case vignettes.

Figure 3 graphs the relative prices of each DRG, compared to the index vignette for the relevant country. The price paid for each DRG for each country studied, is given in Table 4. The names and codes of the DRG each vignette is mapped to, is presented in Additional file 1.

**Table 4:** Vignette prices

		**Austria**^**1**^	**England**^**2**^	**Estonia**^**3**^	**Finland**^**4**^	**France**^**5**^	**Germany**^**6**^	**Ireland**^**7**^	**Poland**	**Spain**^**8**^	**Sweden**^**9**^
Index case	DRG	Mel08.02C	EA14Z	107	107A	05C051	F32Z	F06B	E06	109	107A
	Price (in €)	8,770	12,157	7,459	11,203	10,430	10,761	20,563	4,526	14,421	13,575
Patient 1	DRG	Mel08.02A	EA15Z	106	107B	05C041	F23Z	F05B	E05	107	107B
	Price (in €)	11,479	17,279	11,857	11,227	12,140	13,079	28,391	4,797	19,836	18,253
	Relative value	1.31	1.42	1.59	1.00	1.16	1.22	1.38	1.06	1.38	1.34
Patient 2	DRG	Mel08.03A	EA20Z	106	104A	05C022	F11A	F03Z (Outlier)	E22	104	104A
	Price (in €)	14,908	15,041	11,857	15,109	16,445	17,867	36,184	5,575	22,070	17,798
	Relative value	1.70	1.24	1.59	1.35	1.58	1.66	1.76	1.23	1.53	1.31
Patient 3	DRG	Mel08.02C	EA15Z	106	107C	05C042	F23Z	F05A	E05	546	107C
	Price (in €)	9,396	17,279	11,857	16,531	14,337	13,079	41,648	4,797	25,060	16,570
	Relative value	1.07	1.42	1.59	1.48	1.37	1.22	2.03	1.06	1.74	1.22
Patient 4	DRG	Mel08.03A	EA20Z	105	104B	05C031	F11B	F04B	E22	105	104B
	Price (in €)	14,282	15,041	8,551	21,116	12,654	15,380	24,947	6,070	16,399	23,984
	Relative value	1.63	1.24	1.15	1.88	1.21	1.43	1.21	1.34	1.14	1.77
Patient 5	DRG	Mel08.03A	EA20Z	106	104B	05C022	F11A	F03Z	E22	104	104B
	Price (in €)	14,908	15,041	11,857	21,116	16,445	17,867	57,879	6,211	22,070	23,984
	Relative value	1.70	1.24	1.59	1.88	1.58	1.66	2.81	1.37	1.53	1.77
Patient 6	DRG	Mel08.03A (Outlier)	EA20Z	108	104B	05C031	F11B (Outlier)	F04B (Outlier)	E22	545	104B
	Price (in €)	10,143	15,041	5,268	21,116	12,654	10,890	14,365	5,575	30,433	23,984
	Relative value	1.16	1.24	0.71	1.88	1.21	1.01	0.70	1.23	2.11	1.77

1) Reported values are based on theoretically calculated scores. Actual hospital payment depends on decisions of states, which make use of nationwide DRG scores in different ways.

2) Based on 2008/9 tariff prices and HRG version 3.5. All relevant to-ups are included in the shown tariff. HRG 4 payments could not be determined. Reported figures are a weighted average of payments for elective and emergency admissions, using the ratio of elective and emergency cases in the study period. Elective admissions receive a different tariff from elective cases in many DRGs.

3) Shadow prices were calculated by multiplying cost weights with the national base rates. In actual payment, hospitals are paid through a mix of DRG based payment and fee-for-service. The actual DRG based payment is only 70% of the reported shadow price with the remaining 30% being related to fee-for-service payments.

4) Actual payment varies by type of hospital (i.e. University, central, local hospitals) and hospital district. Provided figures are volume weighted averages across all hospitals. Outlier limits differ between hospital districts. In some hospital districts, patient 3 might be considered an outlier. In this case certain surcharges based on per diems might be applied.

5) Reported prices are for public sector hospitals since private hospital prices do not reflect full costs.

6) Calculated using national DRG cost weights and the average of state-wide base rates (2803.05 €).

7) Calculated using AR-DRG V 5.1 and the national average inpatient 'base price' of €5,219 for a relative value of 1 and the other casemix parameters (including low and high length of stay trim points) from the 2009 inpatient casemix model (used to estimate the 2010 casemix budgetary adjustments on the basis of 2008 activity and cost data).

8) AP-DRGs in Spain are used when patients receive care in non-resident autonomous communities (ACs). The prices shown are the rate that would be paid for these patients, calculated by multiplying national Spanish cost weights with the national base rate. The payment to hospitals for all other patients depends on the hospital payment system in the relevant autonomous community. When using AP-DRGs in Spain, inliers/outliers are not determined.

9) Actual hospital payment depends on the county council, which is free to decide how to pay hospitals.

#### Vignette 1

The additional price for case vignette 1, relative to the index vignette, ranges from 0 (Finland) to 59% (Estonia). In all nations included, with the exceptions of Austria and Poland, patients are grouped based on the occurrence of catheterisation. The Austrian system uses multiple vessels bypassed as a classification variable, though not the use of catheterisation. However, this system includes an additional payment when catheterisation is used. The Polish DRG system does not provide additional payments when catheterisation is used or multiple vessels bypassed. However, an additional 6% is paid if the patient is aged over 69. In Finland, the index vignette and vignette 1 are categorised to different DRGs. However, the difference in reimbursement is minimal (0.002%).

#### Vignette 2

The proportional difference in reimbursement given to vignette 2, relative to the index vignette, ranges from 23% (Poland) to 76% (Ireland). All systems analysed, with the exception of the Estonian, place patients who undergo a valve procedure into a different DRG to those who do not. As noted above, in Estonia, classification differentiates for the use of catheterisation. As such, vignette 1 and 2 are grouped together. The English system categorises valve procedure patients into a broad category of “complex cardiac surgery”, rather than a CABG specific category. The lower relative value of vignette 2 (24%) to vignette 1 (42%), suggests the average cost of this broader group is lower than CABG specific groups. This highlights the sort of disparities which can arise when a small number of DRGs are used to classify heterogeneous patients. In general, a higher tariff is paid for patients undergoing valve procedure with a CABG than catheterisation alone (vignette 1), with the highest tariffs paid by systems which reimburse based on catheterisation and valve procedures (Austria, France, Germany, Ireland and Spain). The French case is slightly unusual as it is the only system analysed which makes some explicit adjustment in tariff for patient death, grouping these with cardiac patients with < 4 days stay in hospital.

#### Vignette 3

The relative price given to case vignette 3, compared to the index vignette, ranges from 6% (Poland) to 103% (Ireland). England, Estonia, Germany and Poland all group vignettes 1 and 3 into the same DRG. The grouping of these patients into the same category is due to these systems not using secondary diagnoses as a split variable or, in the Polish case, not adjusting for comorbidities in patients aged over 70. In both vignettes 1 and 3, patient age is 75. This structural distinction of inclusion or exclusion of comorbidities as a split variable, may partially account for the relatively high premium given in England and Estonia (42% and 59% respectively) for patients with CABG and catheterisation. As comorbidities are expected to increase the cost of treatment, if catheterisation is an indication of greater complexity, patients with comorbidities will be more likely to receive catheterisation than those without. Therefore, the average price of vignettes involving catheterisation will be proportionally larger than the group without catheterisation. If, on the other hand, comorbidities are themselves split variables as well as catheterisation, the increase for catheterisation without comorbidities can be expected to be lower. The most complex cases would not be in this group.

However, it should be noted that a small number of broad categories, doesn’t necessarily result in large differences for catheterisation specifically. The Polish system makes no adjustment for catheterisation and increases reimbursement by 6% on the grounds of age alone. Other drivers of such a difference are the relative homogeneity of the index DRG and the total expected cost range of CABG procedure cases. In Austria and Sweden, a lower tariff is paid for vignette 3 than vignette 1. The critical distinction for the Austrian system is that in vignette 3, a single vessel is bypassed, rather than three in vignette 1. This indicates the Austrian system provides higher reimbursement for multiple vessel bypasses than for secondary diagnoses. By contrast, the Swedish system categorises vignette 3 into a DRG involving bypass and comorbidities, though without catheterisation, indicating catheterisation is of greater weight in that system. In the remaining four countries analysed, a higher tariff is paid for vignette 3 than vignette 1, categorising vignette 3 into a DRG involving comorbidities and providing at least 37% higher tariff than the index vignette.

#### Vignettes 4 and 5

Additional reimbursement for case vignette 4, relative to the index vignette, ranges from 14% (Spain) to 88% (Finland). The increase in reimbursement for vignette 5 ranges from 24% (England) to 181% (Ireland). A comparison between the payments given for case vignettes 2, 4 and 5 illustrates the variety of approaches with respect to the payment for catheterisation, and secondary diagnoses, in the presence of valve procedures. In England and Austria, all three vignettes are grouped into the same DRG, indicating that when a valve procedure is performed, the occurrence of catheterisation or comorbidities does not impact on DRG. As noted above, the Austrian system uses a surcharge to increase a tariff which involves catheterisation. So though the DRG of all the patients is the same, the payment for patients 2 and 5 is higher than patient 4. The Estonian, French, German and Spanish systems pay for catheterisation but not for atrial fibrillation as a secondary diagnosis, grouping vignettes 2 and 5 into a common higher DRG than vignette 4. In Finland and Sweden, a higher tariff is paid in the presence of secondary diagnoses and this tariff is the same with or without catheterisation, grouping vignettes 4 and 5 into a common higher tariffed DRG than vignette 2. In Ireland and Poland, a higher tariff is paid for vignette 5 than either vignette 2 or 4. However, they differ in that the Polish system pays a higher tariff in the case of comorbidities, with or without catheterisation, while the Irish system puts greater value on the presence of catheterisation in calculating reimbursement, while making a smaller adjustment for the case of comorbidities in the absence of catheterisation.

#### Vignette 6

The change in reimbursement for case vignette 6, relative to the index vignette, ranges from −30% (Ireland) to +111% (Spain). Patient 6 combines a relatively complicated case with a short length of stay which ends in death. English, Finnish, French and Swedish systems, place vignette 6 into the same DRG and pay the same amount for this patient as vignette 4. In Austria, Germany, Ireland and Poland, vignettes 4 and 6 are grouped into the same DRG, but pay a lower amount for vignette 6, categorising it as a short stay outlier. As such, this difference in reimbursement is driven by the length of stay rather than the final outcome. However, where death occurs after a CABG procedure, it is also correlated with a relatively short LoS. Therefore, the lower reimbursement may represent an implicit penalty for cases ending in death. The Estonian and Spanish systems place vignette 6 into a different DRG than vignette 4. In Estonia, a lower payment is given for vignette 6. This is driven by diagnosis rather than the presence of death. Also in Spain there is no adjustment for the case of death but a higher tariff is paid to account for the complexity of the case, categorising the patient into a major CC DRG. It is noteworthy from this vignette that while only France explicitly categorises patients based on outcome, where occurrence of death is associated with a short LoS, a short stay outlier can simultaneously represent a lower payment for less complex patients and the same reduction for patients who died in hospital as a penalty for poor outcome.

## Discussion

The definition of patient categories and their weights are two major sets of decisions made in the construction or updating of a DRG system. This study presents the degree of variation in past choices made across Europe. The analysis reveals the wide variation that persists in the number of categories patients are grouped into. It should also be noted that having a larger number of groups does not necessarily result in a more accurate reflection of the variation in cost of CABG patients [10], the variation in the particular variables used to define the boundaries of DRGs and how these are hierarchically structured is also important. The decision as to the weight attached to particular variables is also explored, drawing particular attention to the different ways that the same characteristic can be dealt with and the impact this has on reimbursement. However, while highlighting some of the most important decisions and options in the DRG construction process, not all decisions are covered.

The Austrian system attributes CABG cases to six DRGs, notably adjusting for multiple vessel bypass as well as valve procedure. Of these, valve procedures carry a higher reimbursement. The age split is not picked up by the case vignettes as it applies to patients younger (and older) than 14. This distinction is only used for the simpler CABG procedures without valve procedures or multiple vessel bypass. Alongside this DRG structure, there are supplementary payment mechanisms for the presence of catheterisation and for shorter length of stay. As a result, four of the case vignettes are grouped to the same DRG but three different reimbursement levels are paid.

The English system has the highest concentration of patients in a single DRG at 85%. Also, the vignettes are grouped into the smallest number of DRGs (three). The critical feature of this system is the distinction between a “First CABG” and “Other Complex Cardiac Surgery and Redos”. This set of DRGs acts in a similar way to valve procedure DRGs in other systems, as patients who receive a valve procedure are grouped to this set of DRGs. However, it is not identical as though there is a separate DRG for complex cardiac surgery and redos with catheterisation, cases with valve replacement do not fall into this category. Another feature of this system is that the reimbursement level for catheterisation (1.42) is higher than that for valve procedure (1.24) potentially due to the broader DRG of complex cardiac surgery including some less complex cases, thus reducing its average cost.

In Estonia, CABG patients are grouped into four DRGs. Both catheterisation and valve procedure are used to group patients. CABG and valve procedure are treated separately in the structure with catheterisation as a secondary split for CABG procedures. The case vignettes indicate the presence of catheterisation dominates that of valve procedure in determining DRG as patients are only grouped to the valve DRG in the absence of catheterisation. Also, a higher reimbursement level is given for catheterisation (1.59) than valve procedures (1.16). The separate diagnosis of aortic (valve stenosis) used in vignette 6 also proves significant as this patient is allocated to a separate, lower reimbursed (0.71) DRG.

Finland and Sweden use the same NordDRG structure. This system also separates cases of CABG and valve procedure but in both cases allocates to DRG based on the presence or absence of comorbidities as well as catheterisation. These systems treat valve procedure as the dominant characteristic, grouping all patients with that procedure outside the DRGs for CABG alone and making separate adjustment for complications and not catheterisation. It is also noteworthy that even though the same NordDRG system is used, reimbursement is not identical. In most cases, relative reimbursement levels are higher in Finland than in Sweden. However, when catheterisation is present in the absence of complications or valve procedure, the Swedish system increases reimbursement by 34% compared to less than 1% in Finland.

The French DRG system uses the largest number of groups (sixteen) for CABG cases. The principal division is between CABG and valve procedures, as it is in several systems. Cases involving a valve procedure are grouped outside the set for CABG and further adjustment is made for the use of catheterisation and the presence of comorbidities at different levels. LoS and cases of death are attributed to specific comorbidity levels within each set of these. This approach allows for maximum flexibility as a different level of reimbursement can be given for the same procedure (such as catheterisation) in different circumstances (CABG or valve replacement) instead of giving an average price for all cases or only making an adjustment in a subset of cases. As such, there are separate reimbursement levels for catheterisation, valve procedure and the two combined (at 1.16, 1.26 and 1.59 respectively). The differential cost of comorbidities is also accounted for in some cases, as in comparing vignettes 1 and 3 but not all, as in comparing vignettes 2 and 5.

CABG patients are grouped into three DRGs in the Polish system. The first distinction is between percutaneous valvuloplasty and CABG. Within the CABG groups, a further distinction is made between presence or absence of comorbidities for patients aged 69 and younger. All vignettes including a valve procedure are grouped to the valvuloplasty. In the remaining two vignettes, the additional 6% are paid to account for patient age being 75.

The Irish DRG system groups CABG patients into eight DRGs. The structure is similar to the French, adjusting for the same characteristics and hierarchy. However, only a dichotomy of comorbidity is used and no adjustment is made for catheterisation in the case of valve procedure, though it is if valve procedure is absent. Further, there is an additional DRG for extreme cases in the separate Pre-MDC. Similar to the French system, the use of comorbidities as additional splits within CABG or valve procedure, allows for nuanced reimbursement. For example, a CABG with catheterisation and without valve procedure receives a reimbursement of 1.38 without comorbidities and 2.03 in the presence of sufficient comorbidities or complexity. Further, in the presence of valve procedure and catheterisation, reimbursement is 1.76 in the absence of comorbidities and 2.81 when present.

Cases of CABG in Germany are grouped into 14 DRGs. Besides DRGs in the Pre-MDC for extreme cases, as in the Irish system, DRGs are split into three main groups of interest. These are CABG, valve procedures and CABG with complex procedures. All vignettes including valve procedures are grouped to a valve category, indicating this dominates the presence of CABG. There is a further subdivision for the use of catheterisation, shown by the payment of 1.66 for vignette 2 and 5 compared to 1.43 for vignette 3. The lower payment of 1.01 for vignette 6 is driven by its short length of stay, showing an outlier adjustment. The equal payment made to vignette 1 and 3 at 1.22, shows the adjustment made for the presence of catheterisation in the absence of valve procedure and that there is no further adjustment for comorbidities in this case.

Finally, the Spanish DRG system groups CABG cases into nine DRGs. Here again a Pre-MDC for extreme cases is used. The first consideration in the main system is between the presence or absence of major comorbidities. Among the case vignettes, vignettes 4 and 6 fall into this category. The distinction between CABG and valve procedure is then used and at last catheterisation. This results in the highest reimbursement being paid for cases considered to include major comorbidities and then a further addition where catheterisation is used, specifically 1.74 for vignette 4 and 2.11 for vignette 6. The other four vignettes show a similar level of flexibility in reimbursement for catheterisation (1.38), valve procedure (1.14) or the procedures together (1.53). It is noteworthy that valve procedures receive a smaller increase in reimbursement than catheterisation.

Through case vignette comparisons, this study draws attention to some of the most frequent characteristics used to define DRG boundaries, namely catheterisation, valve procedures and complications. This is not an exhaustive list of the characteristics considered, as shown in the description of patient classification. As such, there is no quantitative comparison of the reimbursement paid for less frequently used characteristics such as the diagnosis of AMI. While these less common characteristics are also of importance, they are less suited for international comparison, as most systems do not differentiate between their presence and absence. Further, the method for paying for extreme outliers is not presented in graphical or numerical analysis. However, this decision is also of critical importance.

The comparison of magnitudes also has limitations. A major obstacle to any cross country comparison is the difference in the purchasing power of currency across nations. By analysing the proportional impact on price of different case vignettes on a common index vignette within each country, this study removes this problem to some extent. However, this approach mitigates, but does not remove, the degree to which different cost categories are accounted for in respective DRG systems. The relative price of a DRG would be exaggerated if the DRG system only pays for variable cost. If instead, some element of fixed cost is included in the tariff, variation will shrink and the value placed on a particular characteristic appear smaller.

Additions within the DRG systems, such as surcharges in Austria for catheterisation, are included in the comparison. However, several idiosyncrasies are not adjusted for in absolute prices. These include adjustments for different regions in England, the summation of multiple DRGs in Finland, and possible separate per diem rates for stays in intensive care units in Austria [13] and Poland [14].

Also, the use of relative difference does not diminish the impact of different coding methods across countries. This is of particular significance in payments for procedures, where common ICD-9-CM codes are mapped to national systems [11]. A national coding system for procedures may also inform the structure of national DRGs.

Finally, this study does not consider the relationship between DRG tariff and the actual cost of a patient. While a DRG system is generally based on past costs of patients, each category groups a set of ultimately unique patients and provides a payment based on some average cost of these. Therefore, the vignette cases serve as examples of different scenarios and the tariff paid for them may not reflect the cost of treatment.

## Conclusions

Despite the limitations of comparing magnitudes across countries, this study highlights the size and complexity of variations between DRG systems across Europe. The decisions which go to make up these systems are of critical importance to clinicians and policy makers. The construction of a DRG system which accurately reflects the cost of best practice, aligns the aims of both sets of stakeholders, and requires the expertise of both to be created and sustained as practice is changed by new findings and innovations. Failure to align these incentives can lead to conflict between the two sets of stakeholders. If the reimbursement for best practice is insufficient to cover its cost or reimbursement for an outdated treatment approach remains the highest available, the patient is liable to receive sub optimal care as the provider is incentivised to maximise revenue.

Whether the variations discussed above are symptomatic of variation in national epidemiological or technological circumstance or the expediency of a DRG system is not the question answered here. Instead, this study presents a set of approaches which have been taken in the past and follows them to their impact upon reimbursement. Without providing evidence as to the optimal structure, the information can be used to suggest alternative approaches that may better align clinical and financial incentives in future system designs or updates. The potential for variation in decisions demonstrates the importance of careful consideration in DRG construction.

## Endnotes

^a^Strictly speaking, in Austria, England and Poland grouping is performed using Patient Classification Systems that are less *diagnosis-related*. However, for convenience and readability we will refer to all systems as DRG systems.

^b^For details regarding the EuroDRG project see http://eurodrg.projects.tu-berlin.de/wiki/doku.php?id=start.

^c^International Classification of Disease 9th edition, clinical modification.

^d^International Statistical Classification of Diseases and Related Health Problems, 10th Revision.

^e^In England and Poland these categories are called Chapters.

## Abbreviations

CABG: Coronary artery bypass graft: 

CC: Complications or comorbidities: 

DRG: Diagnosis related group: 

IHD: Ischemic heart disease: 

LoS: Length of Stay: 

MDC: Major Diagnostic Category: 

OR: Operating room: 

PCCL: Patient Clinical Complexity Level: 

PTCA: Percutaneous Transluminal Coronary Angioplasty: 

## Competing interests

The authors declare that they have no competing interests.

## Additional file

## Supplementary Material

Additional file 1: Vignette names codes. Click here for file
